# Assessment of Two Portable Real-Time Particle Monitors Used in Nanomaterial Workplace Exposure Evaluations

**DOI:** 10.1371/journal.pone.0105769

**Published:** 2014-08-22

**Authors:** Yuewei Liu, Catherine C. Beaucham, Terri A. Pearce, Ziqing Zhuang

**Affiliations:** 1 Institute of Health Surveillance, Analysis and Protection, Hubei Center for Disease Control and Prevention, Wuhan, Hubei, China; 2 National Institute for Occupational Safety and Health, National Personal Protective Technology Laboratory, Pittsburgh, Pennsylvania, United States of America; 3 National Institute for Occupational Safety and Health, Division of Surveillance, Hazard Evaluations, and Field Studies, Cincinnati, Ohio, United States of America; 4 URS Corporation, Pittsburgh, Pennsylvania, United States of America; University of California, Merced, United States of America

## Abstract

**Background:**

Nanoparticle emission assessment technique was developed to semi-quantitatively evaluate nanomaterial exposures and employs a combination of filter based samples and portable real-time particle monitors, including a condensation particle counter (CPC) and an optical particle counter (OPC), to detect nanomaterial releases. This laboratory study evaluated the results from CPC and OPC simultaneously measuring a polydisperse aerosol to assess their variability and accuracy.

**Methods and Results:**

Two CPCs and two OPCs were used to evaluate a polydisperse sodium chloride aerosol within an enclosed chamber. The measurement results for number concentration versus time were compared between paired particle monitors of the same type, and to results from the Scanning Mobility Particle Spectrometer (SMPS) which was widely used to measure concentration of size-specific particles. According to analyses by using the Bland-Altman method, the CPCs displayed a constant mean percent difference of −3.8% (95% agreement limits: −9.1 to 1.6%; range of 95% agreement limit: 10.7%) with the chamber particle concentration below its dynamic upper limit (100,000 particles per cubic centimeter). The mean percent difference increased from −3.4% to −12.0% (range of 95% agreement limits: 7.1%) with increasing particle concentrations that were above the dynamic upper limit. The OPC results showed the percent difference within 15% for measurements in particles with size ranges of 300 to 500 and 500 to 1000 regardless of the particle concentration. Compared with SMPS measurements, the CPC gave a mean percent difference of 22.9% (95% agreement limits: 10.5% to 35.2%); whereas the measurements from OPC were not comparable.

**Conclusions:**

This study demonstrated that CPC and OPC are useful for measuring nanoparticle exposures but the results from an individual monitor should be interpreted based upon the instrument's technical parameters. Future research should challenge these monitors with particles of different sizes, shapes, or composition, to determine measurement comparability and accuracy across various workplace nanomaterials.

## Introduction

A nanoparticle is defined as a nano-object with at least one dimension that is less than 100 nanometers (nm) [1]. Nanoparticles are produced by both natural (incidental nanoparticles) and industrial processes (engineered nanoparticles) [2]. Recent studies have reported the presence of both incidental and engineered nanoparticles in a variety of workplaces [3]–[5], and highlighted their potential adverse health effects [1], [6], [7] which have drawn great concerns for workers producing or utilizing nanotechnology-enabled material [8].

One critical area of study includes measurement methods for nanomaterials. The current methods for measuring the mass of respirable particles in air should be evaluated for their applicability in measuring nanomaterials [9]. No single particle monitor is able to measure the complete size range of interest using a single particle monitor because nanomaterial may be present in a broader range of sizes or larger morphologies. In addition, particle monitors are not able to specifically identify the individual particle size of interest, but can only measure particles in one size range. In 2008, the National Institute for Occupational Safety and Health (NIOSH) nanotechnology field study team developed a nanoparticle emission assessment technique (NEAT), which employed a combination of direct-reading, portable instruments to detect releases of airborne nanomaterial coupled with filter-based air sampling and subsequent chemical and microscopic analyses for particle identification and chemical speciation [4], [10]. This technique has been used to assess potential nanomaterial exposure and the effectiveness of engineering controls in occupational settings [11], [12]. The direct-reading instruments described in the NEAT include two portable real-time aerosol monitors, a condensation particle counter (CPC) which measures in particles per cubic centimeter of air (#/cc) and an optical particle counter (OPC) which measures in particles per liter of air (#/L) [10]. These instruments are used to supplement the filter based sampling, and aid the investigator in determining potential emissions or recommending exposure controls. They are neither used alone to characterize the nanoparticle size distribution nor to determine the particle count of particles in the 10–100 nanometer size range.

Variability between instruments using the same counting principle has not been well studied, and size-specific measurements collected by field-portable instruments such as the CPC have not been well characterized in comparison with laboratory based sizing instruments such as the Scanning Mobility Particle Spectrometer (SMPS). Recent field and laboratory based studies demonstrated that the comparability of real-time particle monitors was not consistent. Compared with results averaged from co-located filter samplers, real-time aerosol monitoring instruments were found to differ between different manufacturers or with different detection technologies, and also instruments of the same model [13]–[16]. If real-time nanoparticle monitoring instruments also demonstrate the same differences, characterizing those differences could be very beneficial for protecting worker health by improving the reliability of exposure assessment. Such studies will also have implications for evaluating workplace protection factors such as those provided by respiratory protective equipment.

In this study, we assessed the variability and accuracy of two of the primary portable real-time particle monitors used in many field exposure assessments, the CPC and OPC. The variability of each monitor was assessed by evaluating the difference of co-located measurements of the same monitor models, whereas the accuracy was evaluated by comparing the measurements of each model with the SMPS.

## Methods

### Experimental Set-up

The set-up established for this study involved a six-jet atomizer (Model 9306; TSI Inc., Shoreview, MN) to generate a polydisperse sodium chloride (NaCl) aerosol in high concentrations with a number mean saline droplet diameter of 350 nm as measured by water. The six-jet atomizer had a built-in pressure regulator and pressure gauge, as well as a self-contained dilution system. One out of the six possible jets was selected. The pressure was set to 30 pounds per square inch (psi) and the NaCl solution was 0.125%. The NaCl aerosol was prepared by creating a 0.125% solution from dry pharmaceutical grade NaCl and deionized water. In addition, the system was supplied with 75 liters per minute (L/min) of dilution air. The aerosol then passed through two 2-millicurie Krypton-85 charge equilibrators (TSI Inc., Shoreview, MN), and then into a large chamber.

The large walk-in chamber had an internal dimension of 1.2 meters (m) ×2.1 m×2.1 m (5292 liters [L]) with 0.75 air changes per hour (Model 222-6; Dynatech Frontier Corp., Albuquerque, NM). The aerosol particles entered the chamber from the top and diffused through holes in the sub-ceiling. A mixing fan assured equal distribution throughout the chamber and chamber pressurization was positive to the laboratory environment.

### Instrumentation

Three different instruments were used in this study. One series 3936 SMPS (TSI Inc., Shoreview, MN) was used. This instrument can classify the mobility diameter of particles from 2.5 nm to 1,000 nm. The SMPS uses a model 3081 Long Differential Mobility Analyzer (DMA) and a model 3080 Electrostatic Classifier (EC) to create a highly monodisperse aerosol. The monodisperse aerosol then passes into a CPC (Model 3776; TSI Inc., Shoreview, MN). This continuous flow counter can detect particles down to 2.5 nm in diameter. It uses real-time coincidence correction and obtains an accuracy of ±10% at <300,000 particles per cubic centimeter (#/cc).

Two portable CPCs (Model 3007; TSI Inc., Shoreview, MN) and two MetOne HHPC-6 Portable Particle Counters (Hach Ultra Analytics Inc., Grants Pass, OR) OPCs were positioned side-by-side in this sample design. The CPC can count particles from 10 to 1000 nm with an upper dynamic range limit of approximately 100,000 #/cc. The CPCs operate by drawing in air at 700 cubic centimeters per minute (cc/min). The accuracy is ±20% and the response time is <9 seconds for 95% response. Counts are displayed in #/cc.

The OPC simultaneously displays six channels of particle size distribution. The size bins used were 300 nm, 500 nm, 700 nm, 1000 nm, 2000 nm, and 5000 nm. Its flow rate is 2.83 L/min. The instrument starts to experience a 5% coincidence loss at 70,000 #/L. The count efficiency is 50% in the 300 nm bin, but 100% for particles larger than 300 nm. Counts are displayed in #/L.

The instruments were either placed inside of the walk-in chamber, or ported into the chamber using a 76.2 cm length of conductive tubing to sample a polydisperse aerosol that was generated to fill the chambers at varied concentrations. The particle concentration in the chamber increased once the atomizer was turned on, and decreased after the atomizer was turned off. Table 1 gives selected parameters for all instruments.

**Table 1:** Specifications of the real-time particle monitors assessed in this study.

Monitors	No. of instruments	Particle size range (nm)	Concentration range (#/cc)	Flow rate (L/min)	Concentration accuracy
SMPS	1	2.5 to 1000, 107 bins	0 to 300,000	0.3±0.015	±10%
Portable monitors					
CPC, model 3007	2	10 to >1000, 1 bin	0 to 100,000	0.70	±20%
OPC, HHPC-6	2	Standard size channel: 300, 500, 700, 1000, 2000, 5000, 6 bins	5% coincidence loss at ∼70,000 #/L	2.83	50% at 0.3 µm; 100% for >0.45 µm

### Data Analysis

Experimental data were analyzed using the SAS software (Version 9.3; SAS Institute Inc., Cary, NC). The difference between each model of portable counter (CPC 1 vs CPC2, OPC 1 vs OPC 2), as well as between SMPS and portable counters was investigated. OPC particle losses due to coincidence error were not considered in the analysis, because a wide range of concentrations were used and there are no specific loss rates for these concentrations. We did not use coefficient of correlation, determination or regression, or compare the means to detect the difference, because these methods have been suggested to be inappropriate ways of assessing agreement between different measurement methods [17]. Instead, we conducted the analyses using the Bland-Altman method, which is extensively used to evaluate the relative agreement between two analytical methods with continuous values on the same scale [18]–[20]. The difference between two instruments' readings as a percentage of their averaged concentration was plotted on the vertical (Y) axis, while the averaged concentration was plotted on the horizontal (X) axis. Mean percent difference and 95% agreement limits (mean percent difference ±1.96*standard deviation [SD]) were calculated and presented in Bland-Altman plots. It is expected that the 95% limits includes 95% of differences between the two measurement methods. A mean percent difference closer to 0 and a smaller range of the agreement limits indicates a better agreement.

We estimated the mean percent difference and its 95% agreement limits according to the method described by Bland and Altman [19]. In brief, we first fitted a linear regression between mean percent difference (D) and averaged concentration of two instruments (A), giving
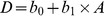
(1.1)
*b_0_* represents the intercept of regression, and *b_1_* represents the slope of regression. If *b_1_* was not significant, we presented the results using a standard Bland-Altman plot with a constant mean percent difference and 95% agreement limits.

If *b_1_* was significant, we obtained the estimated difference between the methods from equation (1.1) for any true value of the measurement, estimated by A, then regress the absolute value of the residuals (R) on A to get
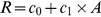
(1.2)
*c_0_* represents the intercept of regression, and *c_1_* represents the slope of regression. If *c_1_* was not significant, the 95% agreement limits were estimated as *b_0_*+*b_1_*×A±1.96× (SD of residuals); or the 95% agreement limits were estimated as *b_0_*+*b_1_*×A±2.46× (*c_0_*+*c_1_*×A).

## Results

**Figure 1:**
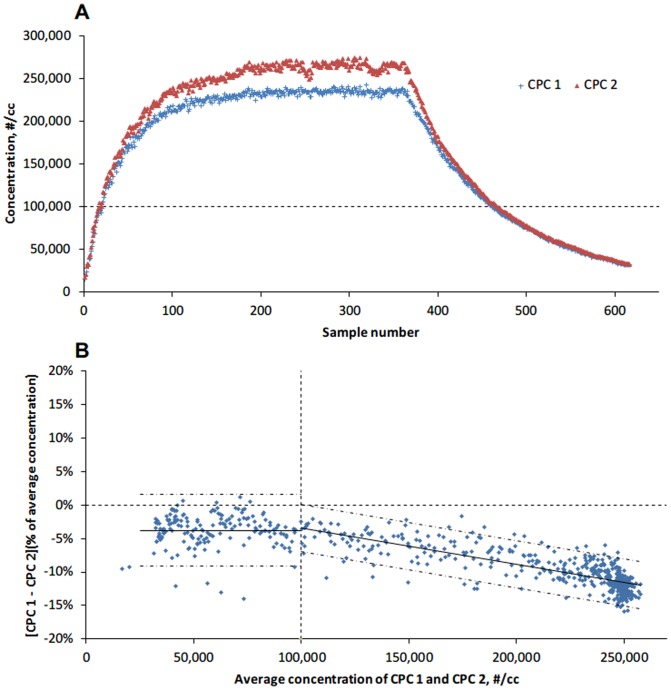
Comparison of two Condensation Particle Counters (CPCs) below and above their dynamic upper limits (100,000 #/cc). The solid lines indicate mean percent difference; the dot-dashed lines indicate ±1.96 standardized deviations (SD) around the mean percent difference; the vertical dashed line represents the CPC's dynamic upper limit; the horizontal dashed line in A represents the CPC's dynamic upper limit; the horizontal dashed line in B represents a percent difference of 0.

The portable CPCs simultaneously measured and data logged 6,167 1-second interval concentrations. A total of 617 pairs of samples were then obtained by averaging concentrations within every 10 seconds. The sample concentrations for CPC 1 and CPC 2 ranged from 15,803 to 242,678 and 17,372 to 274,888 #/cc, respectively. The scatter plot (Figure 1A) showed that the CPCs gave similar results when the chamber concentration was relatively low. Remarkable difference of CPC measurements was observed with higher chamber concentration. For comparison via the Bland-Altman method (Figure 1B), the averaged concentrations of corresponding CPC 1 and CPC 2 samples was divided into 2 levels with a cutpoint of the dynamic upper limit concentration (100,000 #/cc) of the CPC. Comparing CPC 1 with CPC 2, the test showed a constant mean percent difference of −3.8% (95% agreement limits: −9.1% to 1.6%; range of 95% agreement limits: 10.7%), when the particle concentration was lower than 100,000 #/cc, whereas the mean percent difference changed from −3.4% to −12.0% (Linear equation: D = 0.02–5.5×10^−7^×A; 95% upper agreement limit: D = −0.01–5.5×10^−7^×A; 95% lower agreement limit: D = 0.06–5.5×10^−7^×A) with increasing concentrations over 100,000 #/cc; however, the range of 95% agreement became narrower compared that below 100,000 #/cc (from a span of ∼10.7% to a span of ∼7%).

**Figure 2:**
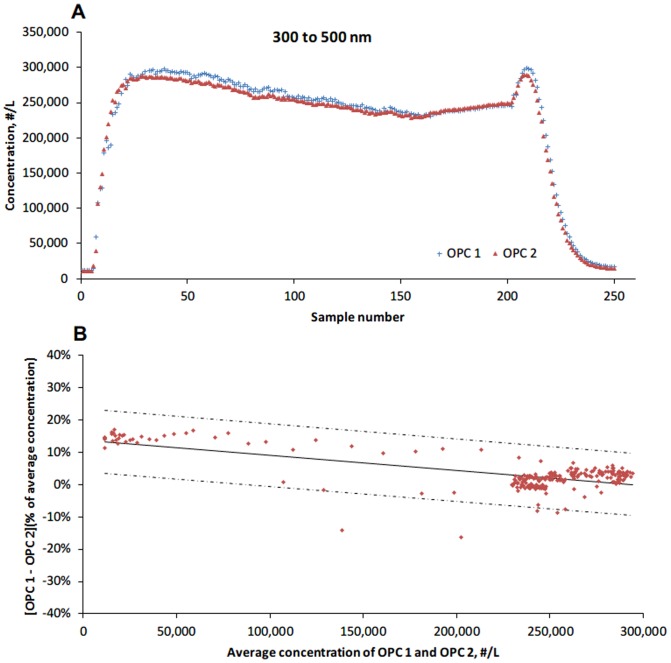
Comparison of 300 to 500 nm particle measurements by two Optical Particle Counters (OPCs). The solid lines indicate mean percent difference; the dot-dashed lines indicate ±1.96 standardized deviations (SD) around the mean percent difference.

**Figure 3:**
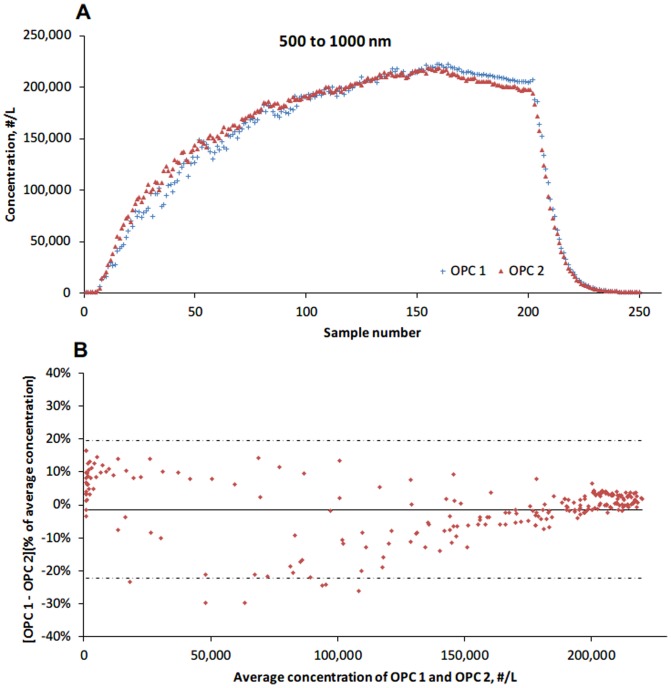
Comparison of 500 to 1000 nm particle measurements by two Optical Particle Counters (OPCs). The solid lines indicate mean percent difference; the dot-dashed lines indicate ±1.96 standardized deviations (SD) around the mean percent difference.

The two OPCs were compared for particles in size ranges of 300 to 500 nm and 500 to 1000 nm. The number concentration for ranges of 500 to 1000 nm was calculated by summing the ranges of 500 to 700 and 700 to 1000 nm. A total of 250 pairs of samples were obtained. The number concentration of 300 to 500 nm particles measured by OPC 1 and OPC 2 ranged from12,100 to 299,327 #/L and 10,701 to 289,757 #/L respectively, whereas the number concentration of 500 to 1000 nm particles measured by OPC 1 and OPC 2 ranged from 883 to 222,312 #/L and 803 to 218,907 #/L. Table 2 represents the mean counts and proportions of each particle size range. Particles with the size range of 300 to 500 nm accounted for over 60% of the total counts. Overall, the two OPCs gave similar measurements across the particle concentration range (Figure 2A). The Bland-Altman (Figure 1B) showed that the mean percent difference decreased from 13.2% to 0.03% (range of 95% agreement limits: 19.3%). As showed in Figure 3, the OPCs measurement differences for 500 to 1000 nm particles were larger, with a range of 95% agreement limits of 41.8% (mean percent difference: −1.4%).

**Table 2:** Mean count and proportion of particle size ranges of 300 to 500 nm and 500 to 1000 nm according to OPC measurements.

	Particle size range, nm			
	All sizes	300 to 500	500 to 1000	>1000
Mean count, #/L				
OPC 1	372,940	224,698	138,256	9,985
OPC 2	370,496	220,281	140,145	10,069
Mean proportion				
OPC 1	100%	65.9%	31.8%	2.3%
OPC 2	100%	65.1%	32.6%	2.3%

**Figure 4:**
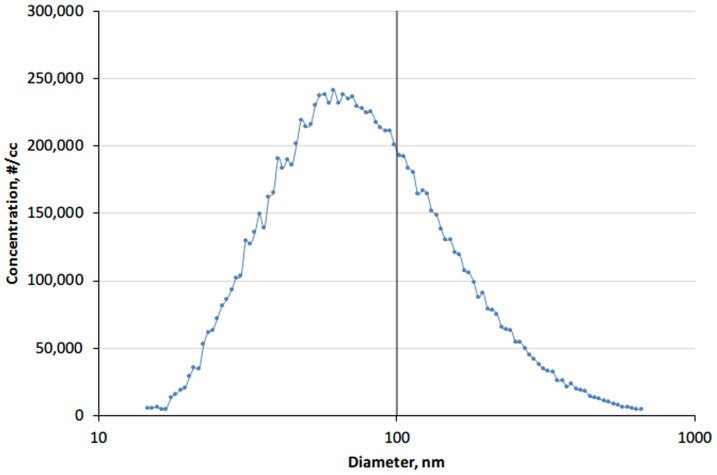
Particle number size distributions of the NaCl aerosol particles measured by SMPS. The vertical solid line indicate a particle diameter of 100 nm.

**Figure 5:**
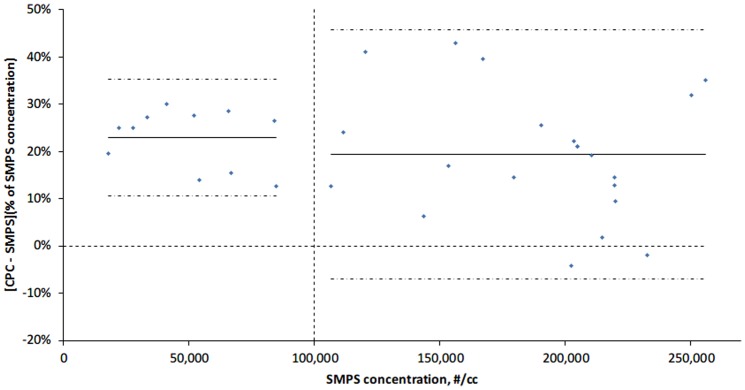
Result of comparing CPC with SMPS. The solid lines indicate the mean percent difference; the dashed lines indicate ±1.96 standardized deviations (SD) around the mean percent difference; the vertical dashed line represents the CPC's dynamic upper limit; the horizontal dashed line represents a percent difference of 0.

Figure 4 shows the particle number size distributions of the NaCl aerosol particles, with a mode size (peak) at ∼62 nm as measured by the SMPS. Figure 5 shows comparison between the portable CPC and SMPS. The CPC measurements reported were obtained by averaging the measurements given by the two CPCs. Compared with measurements by the SMPS, the CPC showed a mean percent difference of 22.9% (95% agreement limits: 10.5% to 35.2%) when the particle concentration was up to 100,000 #/cc. The mean percent difference was 15.4% if the concentration was above 100,000 #/cc, with a wider range of 95% agreement limit (−13.8% to 44.6%). The OPC concentrations were up to 18 times lower and were not comparable to that by the SMPS (data not shown).

We evaluated the count difference method used to determine the number concentration for particles below 300 nm. This was calculated by subtracting the averaged concentration of 300 to 1000 nm particles measured by the OPC from the averaged concentration by the CPC [5], [21]. Additionally, the concentrations of 10 to 300 nm particles were obtained from measurements taken by the SMPS. Comparing the calculated count difference number concentration with the number concentration measured by the SMPS, the mean percent difference for the concentrations estimated by the count difference method was 30.0%, with the 95% agreement limits of 7.6% to 52.9%.

## Discussion

This study demonstrated that the handheld CPCs performed with a reasonable variability (mean percent difference: 3.8%). Data indicated that aerosol concentration may affect performance of the instrument. Compared with measurements obtained with the SMPS, the CPC provided concentrations that were on average 23% higher. The OPC performed with a variability of less than 15% for particles of 300 to 500 nm and 500 to 1000 nm in diameter, regardless of the particle concentration; however, the comparability of OPC and SMPS is poor.

The NEAT recommends using a combination of a CPC and an OPC in conjunction with filter based sample, to determine the particle number concentration and approximate particle size distribution of nanomaterials in the workplace [10]. Based on the NEAT method, the CPC was used to measure particles in the size range of 10 to 1000 nm, though it also responds to larger particles. This instrument is considered particularly useful in that it can detect particles in the 10 to 100 nm range (the current definition of a nanomaterial) [1]. Although the OPC only measures particles in the size range above 300 nm, it was used along with the CPC to provide a differential evaluation of the particles being sampled. For example, a high particle number concentration given by a CPC, along with a high particle count in the 300 to 500 nm range on am OPC, could indicate the potential presence of nanomaterial being sampled; conversely, a low CPC particle number concentration, along with a high OPC particle count in the >1000 nm range would indicate the presence of non-nanosized large particles and/or agglomerates [4]. Measurements obtained by the simultaneous use of both a CPC and an OPC have been used by researchers to calculate particle number concentrations less than 300 nm (referred to as very fine particles) by subtracting concentrations of 300 to 1000 nm particles measured by an OPC from particle concentrations from a CPC (10 to 1000 nm) [5], [21]. It was estimated that this count difference method was able to estimate very fine particle number concentrations with an error between 10.9 to 58.4% [22]. Our results showed similar agreement limits (7.6% to 52.9%) between measurements by the SMPS and the mean difference method in measuring very fine particle number concentrations.

Previous studies suggested that data exceeding the upper dynamic range of the particle counters should be interpreted with caution [4]. The CPC has a dynamic upper range limit of 100,000 #/cc and a variability of ±20%; therefore the range of percent difference between two CPCs was supposed to be within 40%. Our results showed that the two CPCs gave an acceptable percent difference (less than 5%) only when the particle concentration was below its upper dynamic range limit (100,000 #/cc), and the range of 95% agreement limits was 10.7%. In comparison, they did not appear to perform consistently in particles with a concentration exceeding their limit, since the mean percent difference dropped (CPC 1 vs CPC 2) with increasing particle concentration, indicating that the two CPCs did not respond to the higher concentration in the same way. A recent study reported that two CPCs of the same model used in this study showed high comparability with deviation on the order of ±5%, independent of particle sizes, but with a strictly limited upper number concentration [23]. This study used a different method for the comparison between instruments which might produce different agreement estimates.

The OPC HHPC-6 is designed to monitor and verify cleanrooms, test filters and detect particle emission source problems. According to manufacturer's specifications, the HHPC-6 starts to experience a 5% coincidence loss at ∼70,000 #/L. As this is a clean room instrument, most facilities that use this instrument do not obtain particle number concentrations above 70,000 #/L. we evaluated the performance of OPCs in simulated particles with concentrations higher than 70,000 #/L, and found that the mean percent difference between two OPCs was less than 15%. The range of 95% agreement limits was less than 20% for measurements of <1000 nm particles. The results indicated a relatively low variability between two OPC even for measuring particles in concentrations exceeding 70,000 #/L.

We found that exceeding the dynamic limit concentration may also decrease the accuracy of these direct-reading instruments. Compared with measurements by the SMPS, CPC counts overall were 22.9% higher. A possible reason for this difference is that the SMPS measured particles with a range of ∼10 to ∼700 nm, whereas the CPC measured particles in size of 10 to 1000 nm, and that the handheld CPC itself had a ±20% error. Nonetheless, the consistency (range of 95% agreement limits: 25%) was acceptable. In comparison, the range of 95% agreement limits increased to 58% in measuring particles above 100,000 #/cc. The OPC measurements were incomparable with those of the SMPS, probably due to the high particle concentration. The degree of coincidence loss depends on the concentration. The magnitude of the coincidence error increases with the concentration of particles. When high concentrations of particles are present, particles passing through the detector will be closer together, causing overlap and other errors in the processing of the light scattering signals. [24] The OPC might have experienced a much higher coincidence loss (5% at ∼70,000 #/L), giving much lower count numbers. This may overestimate the number concentration of 10 to 300 nm particles when the OPC is used in the count difference method, suggesting that the count difference method should be used with caution and only if the nanomaterial is well characterized and the concentration is lower than 70,000 #/L.

One limitation of our study is that we did conduct the experiment using other aerosols such as titanium dioxide (TiO_2_), silicon dioxide (SiO_2_), or iron oxide (Fe_2_O_3_), because different materials may have different refractive indices and therefore may be measured differently by the portable particle counters. We will continue to compare the results using NaCl with other aerosols in the future experiments. Another limitation is that we did not analyze the potential effect of environmental parameters (such as temperature and humidity) on the performance of the particle counters; however, we tried to maintain the environment in the chamber stable, which at least could minimize effect of the environment variation on counters' performance. Despite of these limitations, the present experiment provided further information as regard to the performance of CPC and OPC in measuring nanoparticles.

## Conclusions

This study suggests that the handheld real-time CPC is useful for measuring nanoparticles, especially the very fine particles (100 to 1000 nm). The OPC can perform consistently but its accuracy may be low when the ambient particle concentration is higher than its dynamic upper limit. The instrument variability should be considered when comparing measurements in different particle monitors. The capabilities may affect the performance of real-time monitors, and should be considered before conducting monitoring and when interpreting results. Future research is necessary for measuring nanoparticles with different sizes, shapes, and composition, and developing field handheld real-time instruments that can measure concentrations and particle size ranges relevant to occupational settings.
